# Cryopreservation of bovine sperm causes single-strand DNA breaks that are localized in the toroidal regions of chromatin

**DOI:** 10.1186/s40104-024-01099-0

**Published:** 2024-10-12

**Authors:** Jordi Ribas-Maynou, Rodrigo Muiño, Carolina Tamargo, Marc Yeste

**Affiliations:** 1 Biotechnology of Animal and Human Reproduction (TechnoSperm), Institute of Food and Agricultural Technology, University of Girona, S17003 Girona, Spain; 2 Unit of Cell Biology, Department of Biology, Faculty of Sciences, University of Girona, S17003 Girona, Spain; 3 Present Address: Unit of Cell Biology and Medical Genetics; Department of Cell Biology, Physiology and Immunology, Autonomous University of Barcelona, S08193 Bellaterra, Spain; 4 Department of Animal Pathology, Faculty of Veterinary Medicine, University of Santiago de Compostela, S15705 Lugo, Spain; 5 Department of Animal Selection and Reproduction, The Regional Agri-Food Research and Development Service of Asturias (SERIDA), S33394 Gijón, Spain; 6 Catalan Institution for Research and Advanced Studies (ICREA), S08010 Barcelona, Spain

**Keywords:** Cattle, DNA damage, Fertility, Sperm, Toroid linker regions

## Abstract

**Background:**

Sperm cryopreservation is widely used in the cattle industry, as it allows for disassociating the localization of sires and the collection of semen from the timing of artificial insemination. While freeze-thawing is known to impair sperm DNA integrity, whether the damage induced consists of single- (SSB) or double-strand breaks (DSB) has not been determined. In addition, no previous study has addressed if DNA breaks preferentially reside in specific genome regions such as those forming the toroid linker regions, or are rather spread throughout the regions linked to protamines. The main aim of the present work, therefore, was to elucidate the type and localization of the DNA damage generated by cryopreservation and to evaluate its impact on artificial insemination outcomes in cattle.

**Results:**

The incidence of SSB and DSB was evaluated in 12 ejaculates before and after cryopreservation with the Comet assay, and the localization of the DNA breaks was assessed using pulsed-field gel electrophoresis (PFGE). Before cryopreservation, the incidence of SSB was 10.99% ± 4.62% and involved 20.56% ± 3.04% of sperm cells, whereas these figures significantly (*P* < 0.0001) increased up to 34.11% ± 3.48% and 53.36% ± 11.00% in frozen-thawed sperm. In contrast, no significant differences in the incidence of DSB were observed (*P* > 0.990) before and after cryopreservation (before: incidence of 13.91% ± 1.75% of sperm DNA affecting 56.04% ± 12.49% of sperm cells; after: incidence of 13.55% ± 1.55% of sperm DNA involving 53.36% ± 11.00% of sperm cells). Moreover, PFGE revealed that the percentage of sperm DNA fragments whose length was shorter than a toroid (< 31.5 kb) was greater (*P* < 0.0001) after (27.00% ± 4.26%) than before freeze-thawing (15.57% ± 4.53%). These differences indicated that the DNA breaks induced by cryopreservation affect the regions condensed in protamines, which are structured in toroids. On the other hand, in vivo fertility rates were associated to the incidence of SSB and DSB in frozen-thawed sperm (*P* = 0.032 and *P* = 0.005), but not with the size of the DNA fragments resulting from these breaks (*P* > 0.05).

**Conclusion:**

Cryopreservation of bovine sperm generates single-strand DNA breaks, which are mainly located in protamine-condensed toroidal regions. The incidence of DNA breaks in cryopreserved sperm has an impact on cattle fertility, regardless of the size of generated fragments.

**Supplementary Information:**

The online version contains supplementary material available at 10.1186/s40104-024-01099-0.

## Introduction

Achieving efficient livestock production is fundamental to ensuring societal access to food and improved profitability for farmers. In this regard, cattle breeding has gone through strong selective pressure to keep the best animals in terms of production, phenotype, and genetic characteristics [1, 2]. Because most of the reproductive strategy for this species is nowadays based on artificial insemination (AI), male gametes need to be stored from ejaculation to insemination. As sperm function and survival cannot be preserved for long-term at temperatures above 0 °C, cryopreservation has been established as the most efficient method and is routinely used in the case of bovine sperm [3, 4]. In spite of the advantages of conducting AI with frozen-thawed sperm, cryopreservation may damage sperm and impair quality parameters, because the male gamete is sensitive to cold shock, osmotic stress and oxidative stress that is associated with cryopreservation procedures, even when permeable and non-permeable cryoprotectants are used. In effect, it has been widely reported that freezing and thawing decrease sperm viability, acrosome integrity, and mitochondrial activity; and increase membrane lipid disorder, DNA damage, and the intracellular levels of reactive oxygen species (ROS) [5, 6]. For this reason, many efforts to ameliorate the resilience of sperm to freeze-thawing and thus improve their post-thaw quality have been attempted over the years. Many of these endeavors have been focused on adding protective molecules to freezing and thawing media, such as those scavenging ROS [7–13].

DNA integrity is one of the most pivotal elements required for embryo progression. Embryos inheriting damaged DNA from gametes exhibit genomic instability, including alterations such as partial chromosome loss or aneuploidies [14–16], which may underlie delays or even arrest preimplantational embryo development [17, 18]. Regarding the paternal inherited genome, DNA breaks have different impact depending if they involve one (single) or the two strands (double) of DNA: whereas the former are associated with fertilization failure the latter are linked to lower blastocyst development rates, respectively [19–22]. Furthermore, while the DNA breaks affecting the toroidal regions condensed in protamines may be widespread and related to oxidative stress, those impairing the toroid linker regions (TLR) are expected to be specifically located and associated to enzymatic activity [23–27].

The success of AI in cattle is not exempt from the sperm DNA damage induced by cryopreservation. Indeed, previous research evaluating 75 cryopreserved ejaculates from 25 bulls found that 25.88% of sperm exhibited a moderate or high degree of DNA damage, thus compromising fertility rates [28]. In agreement with these findings, other studies observed that greater DNA fragmentation in frozen-thawed sperm is related to impaired fertility in this species [29, 30]. Despite this, whether DNA breaks occurring as part of cryodamage are preferentially localized in toroidal or toroid-linker regions has not been determined, nor if they have an impact on cattle reproduction. Furthermore, while earlier investigations consistently demonstrated that bovine sperm cryopreservation is a potential source of sperm DNA fragmentation [31–35], whether it induces single- or double-strand DNA breaks has not been interrogated. On the other hand, because not all sperm exhibit the same resilience to freeze-thawing and differences exist between and within individuals [3, 6, 36, 37], whether variations also occur in the case of sperm DNA damage also warrants further research.

Because of all the aforementioned, the present work aimed to address the type (single- or double-strand) of the sperm DNA damage caused by cryopreservation, as well as the size of the resulting fragments to infer if these breaks spread throughout the genome or are confined in specific regions (i.e., in toroids, or in toroid-linker regions). This study also investigated if the sperm DNA fragmentation induced by cryopreservation occurs concomitantly with alterations in other sperm functional parameters, such as production of intracellular ROX, and also addressed its impact on their fertilizing capacity.

## Materials and methods

Unless otherwise stated, all reagents were purchased from Sigma-Aldrich (Saint-Louis, MO, USA).

### Animals and semen collection

Twelve ejaculates coming from different sexually mature (1.5 to 2 years) bulls from 2 different breeds, 7 Holstein bulls and 5 Asturiana de los Valles bulls, were used. Semen volume obtained per ejaculate was 7.91 ± 2.54 mL and sperm concentration was 1,256.08 × 10^6^ ± 318.20 × 10^6^ sperm/mL. Animals were housed at the Cenero Artificial Insemination Centre (Gijón, Spain) and were handled in fulfillment of the standardized methods for animal husbandry, complying with European Regulations. As samples included in the present study were collected for commercial purposes and the authors did not make any intervention, it did not require the approval of an Ethics Committee. For collection, which was conducted by specialized technicians, bulls were first stimulated by false mounting, and semen samples were then harvested in an artificial vagina which maintained its internal temperature at 45 °C.

### Preparation of sperm samples

Sperm were diluted in BioXcell medium (IMV Technologies, L’Aigle, France) at 20 °C to a concentration of 92 × 10^6^ sperm/mL, as determined with a SDM4 photometer (Minitüb, Tiefenbach, Germany). Samples were then cooled to 5 °C for 90 min (at a cooling rate of approximately 0.2 °C/min), and then held at 5 °C for further 150 min. Thereafter, each semen sample was split into two fractions: one was transported to the laboratory at 5 °C and subjected to analysis (chilled semen), and the other was cryopreserved following the procedure published before [38]. Briefly, sperm were loaded into 0.25-mL straws (23 × 10^6^ sperm per straw) before straws were placed inside a controlled-rate programmable freezer (Digitcool; IMV Technologies, L’Aigle, France). Straws were cryopreserved as follows: 5 °C/min from 4 °C to −10 °C; 40 °C/min from −10 °C to −100 °C; and 20 °C/min from −100 °C to −140 °C. Upon completion of cryopreservation, straws were stored in a liquid nitrogen tank.

When needed for analysis or AI, straws were thawed at 38 °C for 40 s. In the case of sperm analyses, three straws from the same ejaculate were simultaneously thawed and their contents pooled in a 5-mL tube. Thawed sperm were incubated at 38 °C in the dark for 120 min. Straws intended for AI were used individually, and conception rates were recorded as described below.

### Analysis of single- and double-strand DNA breaks: alkaline and neutral Comet assays

Incidences of single- and double-strand DNA breaks were determined before (chilled semen) and after cryopreservation (frozen-thawed sperm) with the Comet assay. The neutral variant of the assay was used to determine the incidence of double-strand breaks, whereas the alkaline one allowed for the evaluation of global DNA damage, which included both single- and double-strand breaks. The protocol followed, which was published before [28], included the steps described below.

#### Embedding of samples in agarose and lysis

First, sperm samples were adjusted to 5 × 10^5^ sperm/mL and then mixed 1:2 (v:v) with 1% low melting point agarose previously molten at 37 °C; the final concentration of agarose was 0.66%. Two 6.5-µL drops of the mixture were immediately placed onto two agarose-pretreated slides (one for the alkaline Comet and the other for the neutral Comet), covered with a round coverslip, and cooled at 4 °C for 5 min. After gently removing the coverslips, each slide was sequentially incubated in three lysis solutions: (i) lysis solution 1, containing 0.8 mol/L Tris–HCl, 0.8 mol/L DTT and 1% SDS (pH 7.5), for 30 min; (ii) lysis solution 2, containing 0.4 mol/L Tris–HCl, 0.4 mol/L DTT, 50 mmol/L EDTA, 2 mol/L NaCl and 1% Tween20 (pH 7.5), for 30 min; and (iii) lysis solution 3, containing 0.4 mol/L Tris–HCl, 0.4 mol/L DTT, 50 mmol/L EDTA, 2 mmol/L NaCl, 1% Tween20 and 100 μg/mL proteinase K (pH 7.5), for 180 min. Following this, slides were rinsed in distilled water.

#### Electrophoresis, neutralization and dehydration

Electrophoresis conditions differed between the slide designated for alkaline Comet and the one intended for neutral Comet. For alkaline Comet, samples were denatured in 0.03 mol/L NaOH and 1 mol/L NaCl at 4 °C for 5 min, before running electrophoresis in a 0.03 mol/L NaOH buffer (pH 13) at 1 V/cm for 4 min. For neutral Comet, samples were directly electrophoresed in a TBE buffer (90 mol/L Tris-base, 90 mmol/L boric acid and 2 mmol/L EDTA; pH 8) at 1 V/cm for 4 min. After electrophoresis, slides subjected to alkaline and neutral Comet were treated equally. First, they were neutralized in a 0.4 mol/L Tris–HCl solution (pH 7.5) for 5 min, and subsequently dehydrated with an increasing ethanol series (70%, 90% and 100% ethanol; 2 min per step). Finally, slides were placed in horizontal position and allowed to dry.

#### Staining, imaging and computerized analysis

Staining was conducted by incubating slides in 25 μmol/L SYTOX Orange (ThermoFisher Scientific, Waltham, MA, USA) at room temperature in the dark for 15 min. Slides were thereupon washed in distilled water at room temperature in the dark for 10 min, placed horizontally, and allowed to dry.

Comet slides were visualized under an epifluorescence microscope (Zeiss Imager Z1, Carl Zeiss AG, Oberkochen, Germany). One hundred fifty sperm were imaged at 100× magnification with Axiovision 4.6 software (Carl Zeiss AG, Oberkochen, Germany), adjusting gain and exposure time in each field to avoid the overexposure of cells. Using these images, the fluorescence intensity of Comet heads and tails in each cell was evaluated through the CometScore v2.0 open-source software (Rexhoover, http://www.rexhoover.com). After utilizing the automatic analysis function, a thorough review of each analyzed cell was performed to eliminate the captures that did not correspond to comets, remove overlapping comets, and correct the misidentifications of Comet heads and tails. A threshold of 80 analyzable comets was set to evaluate the degree of DNA damage.

#### Determination of the percentages of sperm with DNA damage, and the extent of that damage

Data from each cell, including Comet head intensity (arbitrary units), Comet tail intensity (arbitrary units) and Comet tail length (pixels), were extracted in .csv format. Microsoft Excel (Microsoft, Redmond, WA, USA) was employed to determine the percentage of DNA in the Comet Tail (%Tail DNA), defined as the percentage of DNA that migrated to the tail, calculated as [Comet tail intensity/(Comet head intensity + Comet tail intensity)] × 100. The value of %Tail DNA of neutral Comet was utilized as a measure of the incidence of double-strand DNA breaks. For the determination of the incidence of single-strand DNA breaks, the %Tail DNA of neutral Comet was subtracted from that of the alkaline Comet.

#### Calculation of percentages of sperm with DNA damage

Percentages of sperm with SSB + DSB, and percentages of sperm with only DSB were calculated through Principal Component Analysis (PCA), which was run separately for alkaline Comet and neutral Comet. First, as Tail DNA and Tail Length are independent parameters indicative of DNA damage in the Comet assay, they were both used as input variables for a PCA. The regression scores obtained in the PCA for alkaline and neutral Comet were then subjected to a two-step cluster analysis based on the between-groups linkage method and Euclidean distance. The clusters identified were in concordance with previous studies [28]: (a) 3 sperm subpopulations that differed in the incidence of single-strand DNA breaks (high, moderate, and low incidence); and (b) 2 sperm subpopulations that differed in the incidence of double-strand DNA breaks (low and high incidence). Regression scores obtained for each sperm subpopulation are shown in Additional file 1 (Fig. S1).

### Determination of DNA fragment sizes in sperm with damaged DNA

Pulsed-field gel electrophoresis was run to elucidate whether sperm DNA breaks induced by cryopreservation were localized in toroidal (associated with protamines) or toroid-linker regions (associated with histones).

#### Sample preparation

Sperm were first treated to generate plugs with lysed DNA. Briefly, samples were enriched with sperm cells (600 × 10^6^ sperm/mL) through centrifugation at 600 × *g* for 5 min. The resulting samples were subsequently mixed 1:1 (v:v) with 2% low melting point agarose, previously liquefied and warmed at 37 °C, and immediately poured into CHEF plug molds (BioRad, Hercules, CA, USA), where the mixture was allowed to jellify at 4 °C for 15 min. Once jellified, plugs containing sperm were incubated with a lysis solution containing 10 mol/L Tris–HCl, 10 mol/L EDTA, 100 mmol/L NaCl, 20 mmol/L dithiothreitol (DTT), 2% sodium dodecyl sulfate (SDS) and 20 mg/mL proteinase K (pH 8.0) at 53 °C for 60 min. After incubation, plugs were cooled at 4 °C for 10 min and then washed twice in 1× TBE buffer.

#### Pulsed-field gel electrophoresis (PFGE)

Pulsed-field gel electrophoresis (PFGE), which was utilized to determine the amount of DNA fragments longer than 15 kb, was adapted to bovine sperm from a previous protocol [27]. In brief, a 1% pulsed-field agarose gel was prepared in a gel caster tray using 0.5 × TBE buffer (0.445 mol/L Tris–HCl, 0.445 mol/L boric acid and 0.01 mol/L EDTA; pH 8) and pulsed-field certified agarose (BioRad, Hercules, CA, USA). The gel also contained 1 × SYBR safe DNA gel stain (ThermoFisher Scientific, Waltham, MA, USA) for further visualization under UV light.

Plugs were carefully placed into wells, and PFGE was conducted in a contour-clamped homogeneous electric field apparatus (CHEF-DR III Variable Angle System, BioRad, Hercules, CA, USA) containing circulating TBE buffer, at 14 °C for 27 h. PFGE was run at a voltage of 4 V/cm and an angle variation of 120°, with initial and final pulse switch times of 6.7 and 33.7 s, respectively. The MidRange FPG Marker (New England Biolabs; Ipswich, MA, USA) was used as a DNA ladder, allowing for the differentiation of DNA fragment sizes (from 15 to 194 kb). Gels were observed under UV using the GelDoc System (BioRad; Hercules, CA, USA).

#### Data analysis

Image Studio Lite (LI-COR Biosciences, Lincoln, NE, USA) was used to quantify the fluorescence intensity of the DNA contained in each lane. Additionally, each lane was divided based on the DNA ladder into different sections to calculate the percentage of DNA fragments with specific lengths. These regions were chosen to address whether the size of the fragments generated by DNA breaks matched with that of toroids (48 kb, or multiples of 48 kb), as potentially occurring in TLR; or it corresponded to a size smaller than a toroid (< 48 kb), thus suggesting that happened at both toroid and TLR. Six categories in terms of fragment size were distinguished: (i) < 31.5 kb; (ii) between 31.5 and 72 kb; (iii) between 72.5 and 121 kb; (iv) between 121.25 and 169.5 kb; (v) between 169.75 and 218 kb; and (vi) > 218 kb. In every lane, the percentage of DNA fragments corresponding to each category was obtained by subtracting the mean background from the mean fluorescence intensity. The result was subsequently divided by the total fluorescence of the lane and multiplied by 100.

### Computer-assisted sperm analysis

Sperm motility and kinematics were evaluated before and after cryopreservation through a computer-assisted analysis system (ISAS, Integrated Sperm Analysis System; Proiser S.L., Valencia, Spain). Briefly, 5 μL of each sperm sample was loaded into a 20-μm Leja chamber slide (Leja Products BV; Nieuw-Vennep, The Netherlands), previously warmed at 38 °C. At least 1,000 sperm per sample were evaluated under a phase-contrast microscope (Olympus BX41; Olympus, Tokyo, Japan) at 100× magnification. CASA settings were those predefined for bull sperm analysis, with particle area being set between 10 and 80 μm^2^. For each sperm cell, the following kinematic parameters were recorded: curvilinear velocity (VCL, μm/s); straight-line velocity (VSL, μm/s); average path velocity (VAP, μm/s); linearity (LIN = VSL/VCL × 100, %); straightness (STR = VSL/VAP × 100, %); oscillation (WOB = VAP/VCL × 100, %); lateral head displacement (ALH, μm); and frequency of head displacement (BCF, Hz). The percentages of motile sperm and those with progressive motility were also determined. Sperm were considered motile when their VAP was ≥ 10 μm/s, and progressively motile when their STR was ≥ 70%.

### Flow cytometry

Sperm viability, acrosome integrity, membrane lipid disorder, mitochondrial activity, and intracellular levels of ROS and calcium were assessed before and after cryopreservation with a CytoFLEX flow cytometer (Beckman Coulter, Fullerton, CA, USA) using 96-well plates. This device was equipped with three lasers (405, 488, and 637 nm), and fluorescent gain was calibrated daily using the Cytoflex Daily QC Fluorospheres (Beckman Coulter, Fullerton, CA, USA). Staining with the corresponding concentrations of each fluorochrome was conducted in 200 μL of PBS containing a final concentration of 1 × 10^6^ sperm/mL. In all cases, 10,000 sperm per sample were examined at a flow rate between 10 and 60 μL/s. Dot-plot analysis was performed with CytExpert Software (Beckman Coulter; Fullerton, CA, USA). Doublets were excluded from the analysis by gating only those particles with similar FSC-A and FSC-H values. After gating for singlets, the flame-shaped corresponding to sperm cells was gated in the FSC-A/SSC-A dot-plot.

All fluorochromes were purchased from ThermoFisher Scientific (Waltham, MA, USA). After staining, sperm were excited with a blue laser (488 nm), and fluorescent signals were detected either with PE channel (585/42), in the case of hydroethidine (HE); FITC channel (525/40), for SYBR-14, YO-PRO-1, Fluo-4, and H_2_DCFDA; or PC5.5 channel (690/50), in the case of Propidium Iodide (PI). In the case of Live Dead Far Red fluorochrome (LD), it was excited with a red laser (488 nm), and the signal was detected with the APC channel (660/10). The specific staining method is described in the following paragraphs.

Sperm viability was evaluated after incubation with SYBR-14 (32 nmol/L) and PI (7.5 μmol/L) at 38 °C in the dark for 15 min. Sperm that were SYBR-14^+^/PI^−^ were considered viable. In this and other staining protocols (PNA/PI, M540/YO-PRO-1, H_2_DCFDA/PI, HE/YO-PRO-1, JC-1/LD, and Fluo4/PI), the percentages of all sperm subpopulations were recalculated after subtracting the percentage of debris particles determined with the SYBR-14/PI staining from the double-negative subpopulation (i.e., SYBR-14^−^/PI^−^).

Acrosome integrity was determined after incubation with 1.2 μmol/L fluorescein-conjugated peanut agglutinin (PNA-FITC) and 5.6 μmol/L PI, at 38 °C in the dark for 5 min. As sperm were not permeabilized, the double-negative sperm subpopulation (PNA^−^/PI^−^) was considered as viable sperm with an intact acrosome.

Plasma membrane lipid disorder was characterized using the combination of merocyanine 540 (M540) and YO-PRO-1, following the protocol described by Rathi et al. [39] and adapted to bovine sperm in our laboratory. Briefly, semen samples were incubated with 2.5 μmol/L M540 and 25 nmol/L YO-PRO-1 at 38 °C in the dark for 10 min. Sperm positive for M540 (M540^+^/YO-PRO-1^−^ and M540^+^/YO-PRO-1^+^) corresponded to those with high lipid disorder in their membranes, and those negative for both M540 and YO-PRO-1 were considered viable with low lipid disorder in their membranes.

Total ROS levels in sperm were measured after staining with 2′,7′-dichlorodihydrofluorescein diacetate (H_2_DCFDA), which reacts with ROS and converts into dichlorofluorescein (DCF) emitting green fluorescence. Sperm were incubated with 100 μmol/L H_2_DCFDA and 5.6 μmol/L PI at 38 °C in the dark for 20 min. Sperm subpopulations positive for DCF (DCF^+^/PI^−^ and DCF^+^/PI^+^) were considered to present high ROS levels, and those negative for both DCF and PI corresponded to viable sperm with low ROS levels.

Superoxide radical levels were evaluated with hydroethidine (HE), which oxidizes to ethidium (E) in the presence of O_2_^•−^. In brief, samples were incubated with 5 μmol/L HE and 31.25 nmol/L YO-PRO-1 at 38 °C in the dark for 20 min. Fluorescence spillover between FITC and PE channels was compensated (2.24% and 7.5%, respectively). Subpopulations positive for E^+^ (E^+^/YO-PRO-1^−^ and E^+^/YO-PRO-1^+^) were recorded as sperm with high levels of superoxides, and those negative for both E and YO-PRO-1 corresponded to viable sperm with low levels of superoxides.

Intracellular calcium levels were assessed after Fluo4 staining, which emits green fluorescence when binds to calcium [40]. Sperm were incubated with 1.17 μmol/L Fluo4 and 5.6 μmol/L PI at 38 °C in the dark for 10 min. Sperm positive for Fluo4 (Fluo4^+^/PI^−^ and Fluo4^+^/PI^+^) were considered as those with high levels of intracellular calcium, and those negative for both Fluo4 and PI corresponded to viable sperm with low levels of intracellular calcium.

Mitochondrial membrane potential was measured following staining with 5,5′,6,6′-tetrachloro-1,1′,3,3′-tetraethylbenzimidazolylcarbocyanine iodide (JC-1) in combination to Live/Dead Far Red staining (LD). JC-1 aggregates when mitochondrial membrane potential is elevated, emitting orange fluorescence [41]. In order to conduct the staining, LD was first diluted 1:285 (v:v) in DMSO. Then, sperm were mixed with 750 nmol/L JC-1 and 0.25 μL of the diluted LD in a total volume of 100 μL and incubated at 38 °C in the dark for 30 min. Sperm stained in orange (JC-1_agg_) were considered as those with high mitochondrial membrane activity, and sperm that were positive for LD were considered non-viable.

### Artificial insemination

To minimize the effect of female factors that could exert a bias, only artificial inseminations conducted throughout the year in unsynchronized heifers with a natural cycle were included. A single artificial insemination was conducted per cycle using frozen-thawed sperm from one straw (0.25 mL, 23 × 10^6^ sperm per straw). Those females that returned to estrus received a second AI using another straw from the same bull. If, after this second AI, animals returned to estrus, they were excluded from the study to avoid that unknown female factors biased the influence of sperm. In this latter case, the follow-up of the animal was lost.

Fertility rates were calculated as the percentage of females that, after being inseminated with frozen-thawed sperm from the same bull, did not return to estrus after 90 days of AI (NRR_90_). Following the conventional procedure of the farm, diagnosis of estrus was made based on the standing heat of females, or secondary estrus symptoms (vulva swelling and reddening, and mucus discharge), without ultrasonography. The total number of artificial inseminations was 6,818, and the average number of inseminations per bull was 568 (minimum 270; maximum: 2,078).

### Statistical analyses

Statistical analyses were conducted with IBM SPSS for Windows ver. 27 (IBM Corp., Armonk, NY, USA), and graphs were elaborated with GraphPad Prism ver. 8 (GraphPad Software, La Jolla, CA, USA). First, normal distribution and homogeneity of variances were evaluated using Shapiro–Wilk and Levene tests, respectively. As variables did not fit with parametric assumptions, non-parametric tests were used for further statistical tests. The Wilcoxon test was used to compare the different variables before and after freeze-thawing. Correlations between sperm variables and fertility rates were calculated with the Spearman test. Multiple linear regression models (using the least squares method computed through a backward method) were run to determine the effects of sperm DNA damage (independent variables) on fertility rates (dependent variable). Two models to predict fertility rates were computed using the following variables measured in frozen-hawed sperm: the first one included the incidence of DSB, the incidence of SSB, and total sperm motility and viability; and the second one included the incidence of each DNA fragment size assessed through PFGE, total sperm motility and viability. For all tests, the level of statistical significance was set at *P* ≤ 0.05.

## Results

### Cryopreservation increases the incidence of single-strand DNA breaks

**Fig. 1:**
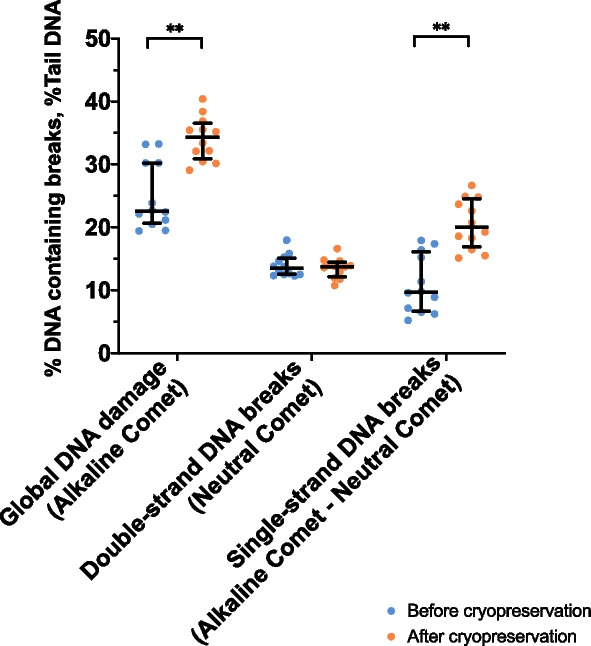
Incidence of DNA breaks before and after cryopreservation. Horizontal lines indicate median values, and whiskers indicate interquartile range. ^**^Double asterisk indicates statistically significant differences compared to chilled semen (*P* < 0.0001)

The Comet assay was conducted before (chilled semen) and after cryopreservation (frozen-thawed sperm) to determine the type and extent of DNA breaks induced by cryopreservation (Fig. 1 and Table 1A). The global incidence of DNA damage, which was measured with the %Tail DNA variable provided by the alkaline Comet, increased significantly (*P* = 0.006) after cryopreservation. In contrast, freeze-thawing did not alter the incidence of double-strand DNA breaks, as evaluated by the %Tail DNA variable of the neutral Comet (*P* = 0.410). Subtraction of the prevalence of double-stranded breaks from that of global DNA damage revealed that cryopreservation significantly increased the incidence of single-strand DNA breaks (*P* = 0.010).

**Table 1:** Incidence of DNA breaks in sperm cells, and percentages of sperm with fragmented DNA in semen samples

Item	**Before cryopreservation** (mean ± SD)	**After cryopreservation** (mean ± SD)	***P*-value**
A) Incidence of DNA breaks			
Global DNA damage, Alkaline Comet Tail DNA	24.90 ± 5.28	34.11 ± 3.48^*^	0.006
Double-strand breaks, Neutral Comet Tail DNA	13.91 ± 1.75	13.55 ± 1.55	0.410
Single-strand breaks, Alkaline Comet Tail DNA − Neutral Comet Tail DNA	10.99 ± 4.62	20.56 ± 3.04^*^	0.010
B) Percentage of sperm with DNA damage			
% Sperm with medium global DNA damage	19.51 ± 11.03	41.41 ± 10.10^*^	0.003
% Sperm with high global DNA damage	6.49 ± 7.54	11.95 ± 6.70	0.147
% Sperm with medium + high global DNA damage	26.00 ± 17.21	53.36 ± 11.00^*^	0.004
% Sperm with double-strand breaks	56.04 ± 12.49	56.02 ± 15.74	0.790

*SD* Standard deviation

*Statistically significant differences between chilled (before cryopreservation) and frozenthawed sperm (after cryopreservation) (*P* < 0.01)

**Fig. 2:**
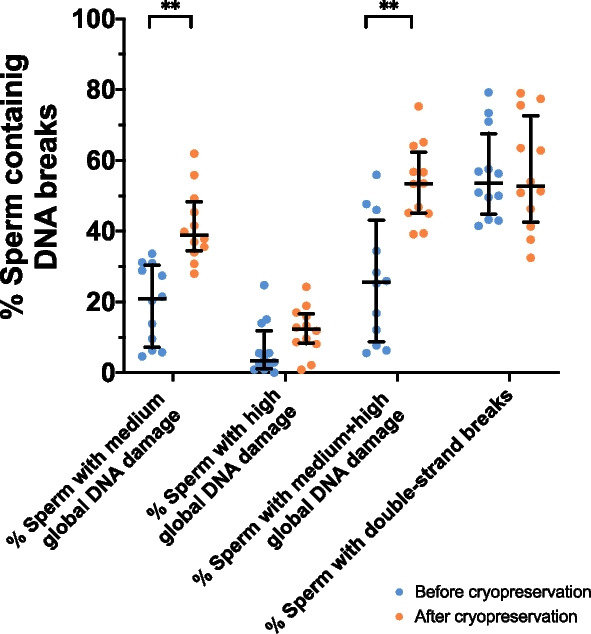
Percentage of sperm containing DNA breaks. Horizontal lines indicate median values, and whiskers indicate interquartile range. **Double asterisk indicates statistically significant differences comparing chilled and frozen-thawed sperm (*P* < 0.0001)

Percentages of sperm with global and double-strand DNA breaks were determined. Whereas three sperm subpopulations, corresponding to low, medium and high incidence of global DNA damage were identified in the alkaline Comet; only two sperm subpopulations, corresponding to low and high incidence of double-strand DNA breaks, were found in the neutral Comet (Table 2). As shown in Table 1B, the percentage of sperm with global DNA damage increased after freeze-thawing (*P* = 0.004). This increase was statistically significant for the sperm subpopulation with medium DNA damage (*P* = 0.003), but not for that with high DNA damage (*P* = 0.147; Fig. 2, Table 1B). In contrast, cryopreservation did not alter the percentage of the sperm subpopulation with a high incidence of double-strand DNA breaks (*P* = 0.790) (Fig. 2, Table 1B).

**Table 2:** Incidence of DNA breaks (Olive Tail Moment, OTM) in the identified sperm subpopulations

Item	Mean ± SD	Median	Minimum	Maximum
Alkaline Comet, OTM				
Low DNA damage	18.14 ± 7.97	17.82	1.26	48.34
Medium DNA damage	38.28 ± 11.99	37.24	8.47	81.98
High DNA damage	72.57 ± 14.73	72.57	31.60	99.93
Neutral Comet, OTM				
Low DNA damage	9.11 ± 2.96	9.55	0.00	15.82
High DNA damage	17.37 ± 7.30	16.21	4.47	98.80

*SD* Standard deviation

### Sperm DNA breaks induced by cryopreservation mainly localize within toroids

**Fig. 3:**
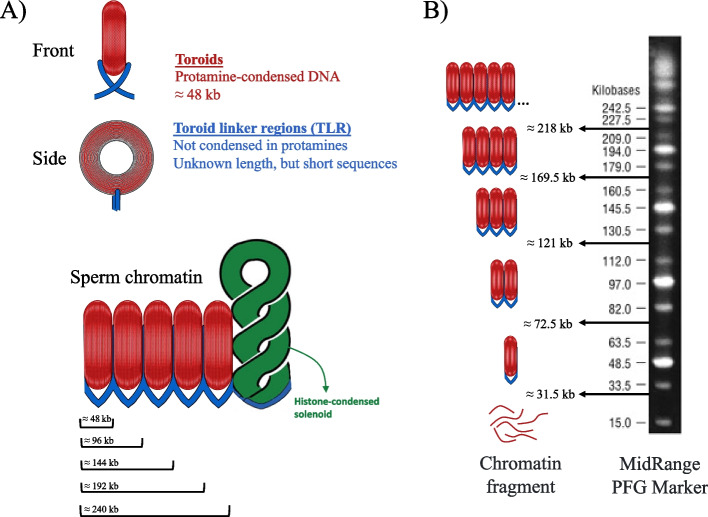
The elements of sperm chromatin structure and the different sections evaluated by PFGE. **A** Diagram showing the different sperm chromatin structural elements: protamine-condensed toroids in red color, toroid linker regions in blue color, and histone-condensed regions in green color. As toroidal regions have been hypothesized to contain about 48 kb of DNA, the length of one to five toroidal sections is shown in the bottom. **B** Image of the MidRange PFG DNA ladder (Catalog #N0342S, New England Biolabs, Ipswich, MA, USA) accompanied with an illustration of the different regions assessed after pulsed-field gel electrophoresis. Each region corresponds to a different amount of toroidal units, or a DNA length shorter than a toroid (< 31.5 kb)

After observing that cryopreservation increased the incidence of single-strand DNA breaks in bovine sperm, whether such breaks were confined to the toroid linker regions or were spread over toroids was interrogated. As the size of toroidal regions is known to be about 48 kb of DNA, the question posed was if the length of the DNA fragments generated by those breaks was a multiple of 48 kb. Figure 3 illustrates the elements of sperm chromatin structure and the different sections evaluated by PFGE.

**Fig. 4:**
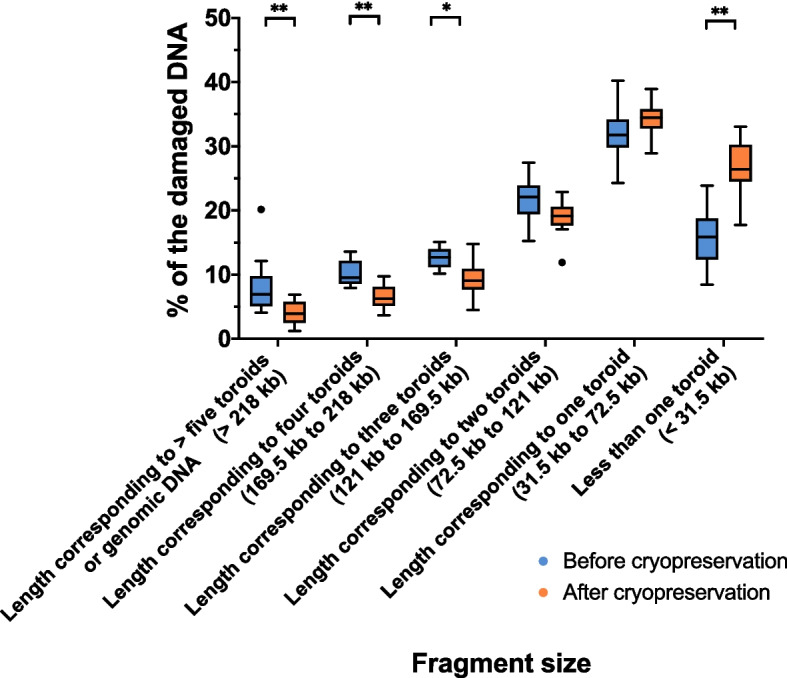
Box and whisker plot showing the distribution of DNA fragments with different lengths. Black dots indicate outlier values. *Indicate statistically significant differences (*P* < 0.05). **Indicate statistically significant differences (*P* < 0.0001)

Figure 4 depicts the length of the different sperm DNA fragments before and after freeze-thawing. Cryopreservation was found to significantly decrease the percentages of DNA fragments longer than 218 kb (*P* = 0.005), those between 169.5 and 218 kb (*P* = 0.005), and those between 121 and 169.5 kb (*P* = 0.012), which all corresponded to long fragments with few breaks. Cryopreservation was also found to increase the percentage of fragments shorter than 31.5 kb (i.e., shorter than the toroid length; before cryopreservation: 15.57% ± 4.53% vs. after cryopreservation: 27.00% ± 4.26%; *P* = 0.003).

### Cryopreservation also alters sperm function and survival

**Fig. 5:**
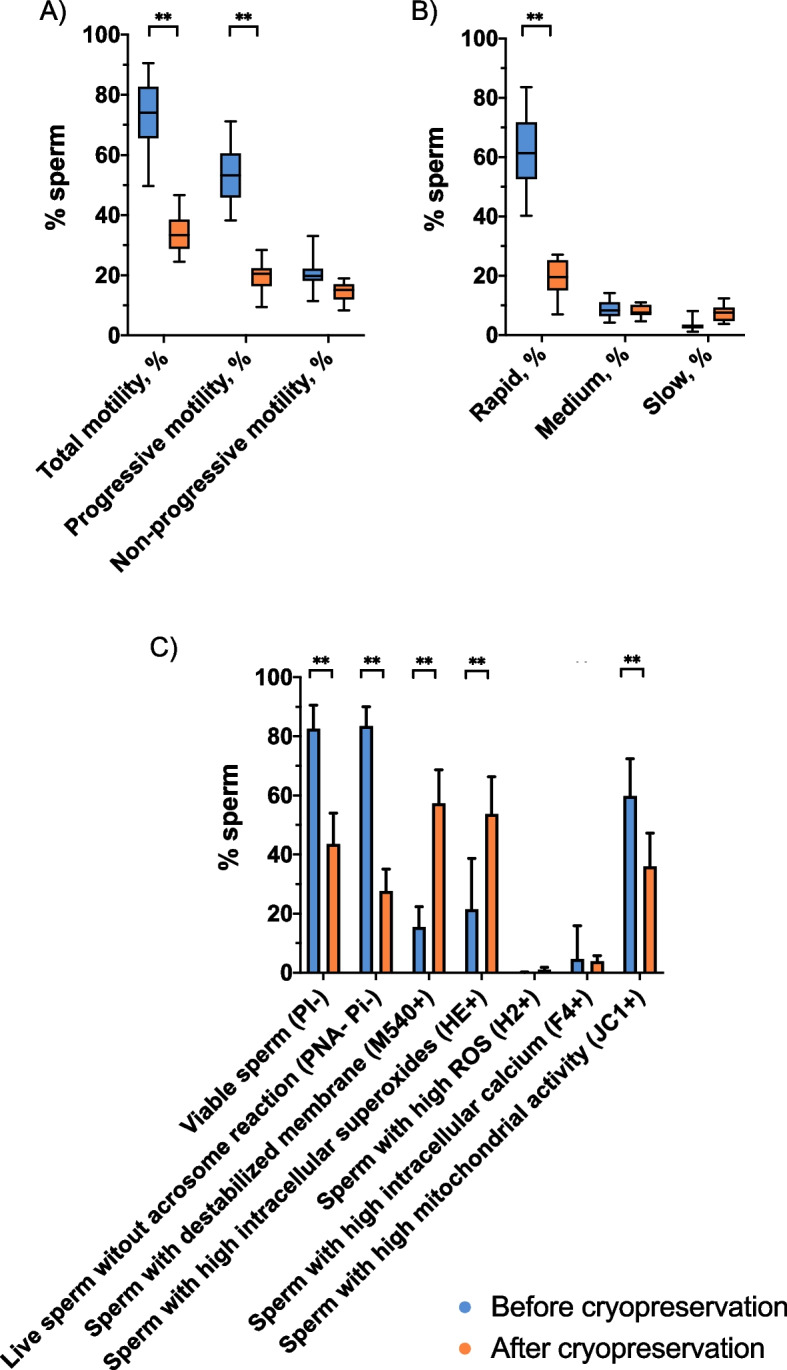
Percentages of sperm for each parameter. **A** Box and whisker plots represent sperm motility parameters. **B** Box and whisker plots show sperm velocity parameters. **C** Bars show the mean and standard deviation for each sperm variable. **Double asterisk indicates statistically significant differences compared to chilled semen (*P* < 0.0001)

As expected, cryopreservation was seen to affect different sperm functional variables, as shown in Table 3, Fig. 5 and Additional file 2. Focusing on ROX, intracellular levels of superoxides (O_2_^•−^) were significantly (*P* = 0.001) higher after (53.11% ± 12.68%) than before freeze-thawing (21.90% ± 16.92%). In addition, total ROS levels evaluated with H_2_DCFDA/PI increased after cryopreservation (before freeze-thawing: 0.11% ± 0.10% vs. after freeze-thawing: 1.05% ± 0.71%; *P* = 0.001).

**Table 3:** Sperm motility, kinetics, and functionality parameters before and after cryopreservation

Sperm motility	**Before cryopreservation** (mean ± SD)	**After cryopreservation** (mean ± SD)	***P*-value**
Total motility, %	73.82 ± 11.56	34.38 ± 6.65^*^	0.001
Progressive motility, %	53.26 ± 9.21	19.86 ± 5.40^*^	0.001
Non-progressive motility, %	20.56 ± 5.59	14.52 ± 3.09	0.004
Sperm kinetic parameters			
Rapid sperm, %	62.02 ± 12.69	19.12 ± 6.42^*^	0.001
Medium sperm, %	8.67 ± 2.91	7.96 ± 2.00	0.650
Slow sperm, %	3.08 ± 1.71	7.29 ± 2.69	0.001
VCL, μm/s	93.72 ± 13.32	72.70 ± 13.16^*^	0.001
VSL, μm/s	53.25 ± 7.88	36.97 ± 7.71^*^	0.001
VAP, μm/s	61.85 ± 9.32	42.26 ± 7.52^*^	0.001
LIN, %	57.18 ± 7.42	50.72 ± 2.87	0.010
STR, %	86.16 ± 3.93	87.05 ± 3.21	0.972
WOB, %	66.22 ± 7.00	58.26 ± 2.37^*^	0.007
ALH, μm	3.18 ± 0.52	3.95 ± 0.37	0.005
BCF, Hz	9.78 ± 0.66	8.67 ± 0.98	0.002
Sperm function			
Viable sperm, % SYBR-14^+^/PI^−^	82.54 ± 7.96	43.51 ± 10.48^*^	0.003
Viable sperm with an intact acrosome, % PNA^−^/PI^−^	83.45 ± 6.59	27.62 ± 7.46^*^	0.003
Sperm with high membrane lipid disorder, % M540^+^	15.44 ± 6.95	57.27 ± 11.45^*^	0.001
Sperm with high intracellular superoxides, % E^+^	21.42 ± 17.22	53.64 ± 12.66^*^	0.001
Sperm with high ROS, % DCF^+^	0.10 ± 0.09	1.06 ± 0.72	0.001
Sperm with high intracellular calcium, % Fluo4^+^	4.64 ± 11.30	3.79 ± 1.94	0.152
Sperm with high mitochondrial activity, % JC-1^+^	59.74 ± 12.66	36.02 ± 11.20^*^	0.001

*SD* Standard deviation

*Statistically significant differences between chilled (before cryopreservation) and frozenthawed sperm (after cryopreservation) (*P* < 0.01)

### DNA damage in bovine sperm impairs fertility rates after artificial insemination, but the length of the resulting DNA fragments is not related to fertilization outcomes

Whether the incidence of DNA breaks, the percentage of sperm with DNA damage, and the localization of these breaks in sperm chromatin have any impact on reproductive outcomes were also examined based on NRR_90d_ after AI with frozen-thawed sperm. Table 4 shows the correlation coefficients and *P*-values between fertility rates and each parameter evaluated after cryopreservation. Among all parameters, the incidence of single-strand DNA breaks in cryopreserved sperm was noticed to exert a negative impact on fertility outcomes (*R* =−0.727; *P* = 0.009) (Table 4A).

**Table 4:** Correlations between sperm chromatin damage variables evaluated after cryopreservation and fertility rates, measured as non-return to estrus rates after 90 days of AI

Item	**Spearman R**	**95% confidence interval**	***P*-value**
A) Incidence of DNA breaks			
Global DNA damage after cryopreservation, Alkaline Comet Tail DNA	−0.531	−0.852 to 0.0802	0.079
Double-strand breaks after cryopreservation, Neutral Comet Tail DNA	−0.573	−0.868 to −0.020	0.055
Single-strand breaks after cryopreservation, Alkaline Comet Tail DNA − Neutral Comet Tail DNA	−0.727^*^	−0.921 to −0.245	0.009
B) Percentage of sperm with DNA damage			
% Frozen-thawed sperm with medium global DNA damage	−0.566	−0.865 to 0.030	0.059
% Frozen-thawed sperm with high global DNA damage	0.202	−0.435 to 0.705	0.528
% Frozen-thawed sperm with medium + high global DNA damage	−0.265	−0.737 to 0.380	0.404
% Frozen-thawed sperm with double-strand breaks	−0.580	−0.870 to −0.009	0.052
C) Percentages of DNA fragments with different length			
Fragments > 218 kb after cryopreservation	0.076	−0.533 to 0.635	0.817
Fragments 169.5 to 218 kb after cryopreservation	−0.398	−0.798 to 0.245	0.201
Fragments 121 to 169.5 kb after cryopreservation	−0.398	−0.798 to 0.245	0.201
Fragments 72.5 to 121 kb after cryopreservation	−0.209	−0.709 to 0.429	0.513
Fragments 31.5 to 72.5 kb after cryopreservation	0.321	−0.326 to 0.764	0.308
Fragments < 31.5 kb after cryopreservation	0.377	−0.268 to 0.789	0.227

*Statistically significant correlations (*P* < 0.01)

On the other hand, none of the categories in which fragment sizes were differentiated (i.e., < 31.5 kb; 31.5 to 72 kb; 72.5 to 121 kb; 21.25 to 169.5 kb; between 169.75 and 218 kb; and > 218 kb) was found to be correlated to fertility rates (*P* > 0.05) (Table 4C).

Multiple linear regression analysis including sperm DNA damage, total motility and viability as independent variables was run to further investigate the association between DNA damage and fertility rates. The first model revealed an association between fertility rates and the incidence of both double-strand and single-strand DNA breaks in frozen-thawed sperm (Table 5A). The second model, however, showed no association between fertility rates and the length of sperm DNA fragments (Table 5B).

**Table 5:** Multiple linear regression models determining the effects of the incidence of DNA breaks (A), and the length of the fragments generated by these DNA breaks (B) on fertility rates

Item	**Parameter estimate (β)**	**95% confidence interval for β**	***P*-value**
A) Model 1: incidence of DNA breaks			
Double-strand breaks after cryopreservation, Neutral Comet Tail DNA	7.938	3.124 to 12.750	0.005^*^
Single-strand breaks after cryopreservation, Alkaline Comet Tail DNA − Neutral Comet Tail DNA	−1.996	−3.779 to −0.212	0.032^*^
Total motility after cryopreservation, %	−0.507	−2.615 to 1.601	0.594
Sperm viability after cryopreservation, %	0.183	−1.081 to 1.448	0.746
B) Model 2: effect of DNA fragment length			
Fragments > 218 kb after cryopreservation	2.156	−9.843 to 14.160	0.644
Fragments 169.5 to 218 kb after cryopreservation	7.888	−13.980 to 29.750	0.373
Fragments 121 to 169.5 kb after cryopreservation	−7.166	−19.890 to 5.563	0.193
Fragments 72.5 to 121 kb after cryopreservation	−3.102	−12.830 to 6.625	0.426
Fragments 31.5 to 72.5 kb after cryopreservation	5.602	−4.5350 to 15.740	0.199
Fragments < 31.5 kb after cryopreservation	−1.027	−8.687 to 6.633	0.728
Total motility after cryopreservation, %	0.361	−4.129 to 4.851	0.834
Sperm viability after cryopreservation, %	−1.184	−3.632 to 1.264	0.250

*The level of significance was set at *P* ≤ 0.05

## Discussion

As sperm quality has unequivocally been identified as one of the main factors contributing to fertility in farm animals, previous research seeking to improve AI outcomes has focused on the evaluation and maintenance of sperm function and survival. Cryopreservation is the best method to store sperm for long-term, and allows for the dissociation between the time and location of semen collection and the occurrence of artificial insemination. In cattle, breeding is mainly achieved through AI with frozen-thawed sperm, notwithstanding cryopreservation is known to impair sperm quality and increase DNA fragmentation. In the present work, cryopreservation was found to raise the incidence of global DNA damage by an average of 9.21%. In addition to this, freeze-thawing was seen to increase the sperm subpopulation having medium or high incidence of global DNA damage. It is worth mentioning that, because bovine sperm only contain protamine 1, complete chromatin decondensation with proteinase K was required before the Comet assay, as examining the extent of DNA damage in the protamine-condensed toroidal regions of those cells is not otherwise possible [42]. This is a critical issue that needs to be considered when one attempts to compare the figures observed in this work with other studies that employed other methods that did not include a previous step of chromatin decondensation, and could thus be less sensitive in the detection of DNA damage [43–46]. This explains why the extent of DNA damage observed herein was greater than the previous literature evaluating the DNA integrity of bovine sperm through Sperm Chromatin Structure Assay (SCSA), Sperm Chromatin Dispersion test (SCD) or TUNEL assay, which found that freeze-thawing increases the percentage of sperm with fragmented DNA from 0.99%–9% (before cryopreservation) to 2%–11% (after cryopreservation) [5, 31, 33, 35]. Only a brief communication by Słowińska et al. [47] evaluated the effects of cryopreservation upon bovine sperm DNA with the Comet assay, and found that freeze-thawing increased %Tail DNA in 3.8%; yet, these authors did not examine the percentages of sperm with fragmented DNA. In a previous work from our research group evaluating another subset of bulls, the percentage of frozen-thawed bovine sperm with moderate or high DNA fragmentation was found to be 25.88%, and the mean alkaline Comet Olive Tail Moment (OTM) was 15.57 ± 5.20 [28]. After checking the raw data from that study, these values of the OTM correspond to a %Tail DNA of 33.66 ± 5.41, which is similar to that obtained here.

Regarding the type of DNA breaks induced by cryopreservation, freeze-thawing was found not to alter the incidence of double-strand DNA breaks and it did increase that of the single-strand ones. While no study previously determined if sperm cryopreservation in bovine increases the incidence of single- and double-strand DNA breaks, the current one concurs with previous research in humans with the Comet assay, where the incidence of single- rather than double-strand DNA breaks was seen to increase after freeze-thawing [48]. Regarding double-strand breaks, the incidence was similar before and after cryopreservation. This value was in agreement with that observed in frozen-thawed bovine sperm by Serafini et al. [49] (%Tail DNA = 15.0% ± 0.5%), and with a previous study from our laboratory, where the reported OTM (1.31 ± 0.20) corresponded to %Tail DNA = 14.50% ± 1.46% [28]. The effects on the incidence of single-strand breaks are discussed in more detail in the next paragraph.

The fact that cryopreservation increased the incidence of single- but not that of double-strand breaks suggests that this type of DNA damage was induced by oxidative stress. This is not surprising, as ROS have been identified as one of the most deleterious disruptors of DNA integrity [50, 51]. In support of this possibility, the present study found that, despite the decrease in the percentage of sperm with high mitochondrial activity, that of sperm with high superoxide levels increased by two-fold after cryopreservation. Superoxides are highly reactive molecules that may be produced in the mitochondria during cell respiration, and can potentially induce base modifications such as 8-hydroxy-2′-deoxyguanosine (8-OHdG), which is cleaved by oxoguanine glycosylase (OGG), the only enzyme of the base excision repair mechanism that is present in sperm and generates a single-strand break [52]. This rise in the percentage of sperm with high superoxide radicals, which has also been reported in the previous literature as a feature of freezing and thawing protocols, could underlie the decline in sperm motility, membrane integrity and mitochondrial activity [33, 53, 54]. Worthy of notice is that seminal plasma, which is a source of enzymatic and non-enzymatic antioxidants that is removed before starting the sperm freezing protocol, plays a crucial role in scavenging ROS. For this reason, several investigations have tested the addition of antioxidants to freezing and thawing media to mitigate the detrimental effects of ROS during cryopreservation [55].

Once established the type and extent of DNA damage, whether the DNA breaks induced by cryopreservation localized in toroidal regions condensed into protamines or in the toroid linker regions was evaluated. Interestingly, cryopreservation was found to increase the percentage of DNA fragments shorter than 31.5 kb, and decreased that of those longer than 218 kb, which were considered as having less damage. As the toroid length is about 48 kb, these data suggest that the single-strand breaks induced by cryopreservation are spread throughout sperm chromatin, and they are, because the greater relative amount, mostly located within the toroids. This possibility perfectly matches with the aforementioned increase in ROS observed after cryopreservation. In effect, ROS are recognized as one of the main effectors of cryoinjury, as oxidant molecules are sterically small and, given the size of toroidal structures, can diffuse throughout the internal regions of sperm nuclei [56–58]. Conversely, nucleases, other DNA cleaving enzymes such as topoisomerases [27, 59], and other DNA damaging effectors like bacterial DNAse activity [32] would not participate in the increase of intratoroidal DNA breaks induced by cryopreservation, as these enzymes are sterically bigger and are unlikely to exert their activity in regions condensed in protamines (toroids) [58].

As a final objective, the impact of sperm DNA fragmentation induced by cryopreservation on cattle reproduction was assessed. Single-strand breaks correlated to non-return rates after artificial insemination, and—as revealed by linear regression analysis—both ssDNA and dsDNA breaks were associated to fertility rates. In previous studies, a high incidence of DNA breaks was found to be linked to embryo development failure [18]. Based on these data, and because cryopreservation was herein observed to increase the incidence of single-strand breaks, one should expect a reduction of fertility rates when DNA damage is greater. Taking into consideration how sire selection operates in the cattle industry, it is reasonable to recommend bull selection based on the low incidence of single-stranded breaks, as this ultimately has an effect on fertilization rates and, thus, on productivity [18, 21]. Finally, no association between fertility rates and the length of fragments resulting from the DNA fragmentation induced by cryopreservation was observed. This suggests that the detrimental effect of DNA damage comes from the breaks themselves rather than from the length of the fragments generated by these breaks. One would thus expect that the alterations in some regions of the genome could be more detrimental for fertility than others, and might also infer that the higher the incidence of global DNA damage, the greater the probability of an important genome region being affected.

## Conclusions

In conclusion, this work demonstrates that, in bovine sperm, cryopreservation increases the extent of DNA fragmentation, mainly consisting of single-strand rather than double-strand DNA breaks. Data also support that oxidative stress could be the main effector underlying the increase in the incidence of cryopreservation-induced DNA breaks. In addition, the study found that these breaks are likely to localize in chromatin toroidal regions, as the size of the fragments resulting from the DNA damage induced by freeze-thawing were shorter than a toroid.

## Supplementary Information

**Additional file 1: Fig. S1.** Representation of the PCA regression scores in each sperm cluster. Left graph shows sperm with low, medium, or high DNA damage for alkaline Comet. Right graph shows sperm with low or high DNA damage for neutral Comet.

**Additional file 2: Table S1.** Sperm function parameters in viable and non-viable sperm, before and after cryopreservation.

## Data Availability

The datasets resulting from the current study are available from the corresponding author at a reasonable request.

## Abbreviations

8-OHdG: 8′-Hydroxy-2′deoxyguanosine
AI: Artificial insemination
ALH: Mean amplitude of lateral head displacement
AU: Arbitrary units
BCF: Frequency of head displacement
DCF: Dichlorofluorescein
DSB: Double-strand DNA breaks
DTT: Dithiothreitol
E: Ethidium
EDTA: Ethylenediaminetetraacetic acid
FITC: Fluorescein isothiocyanate
H_2_DCFDA: 2′,7′-Dichlorodihydrofluorescein diacetate
HE: Hydroethidine
JC-1: 5,5′,6,6′-Tetrachloro-1,1′,3,3′-tetraethylbenzimidazolylcarbocyanine iodide
LIN: Linearity coefficient
NRR: 90-Day non-return rates to estrus
OGG1: 8-Oxoguanosine DNA glycosylase
PCA: Principal Component Analysis
PI: Propidium Iodide
PNA: Fluorescein-conjugated peanut agglutinin
PFGE: Pulsed field gel electrophoresis
ROS: Reactive oxygen species
SCD: Sperm Chromatin Dispersion test
SCSA: Sperm Chromatin Structure Assay
SSB: Single-strand DNA breaks
STR: Straightness coefficient
TUNEL: Terminal deoxynucleotidyl transferase dUTP nick end labeling
TLR: Toroid linker region
VAP: Average path velocity
VCL: Curvilinear velocity
VSL: Straight-line velocity
WOB: Wobble coefficient
