# Supplementary figures and images for: Cryopreservation of bovine sperm causes single-strand DNA breaks that are localized in the toroidal regions of chromatin
Source: J Anim Sci Biotechnol. 2024 Oct 12;15:140. doi: 10.1186/s40104-024-01099-0 (PMC11470689; doi:10.1186/s40104-024-01099-0)

**Alkaline Comet**

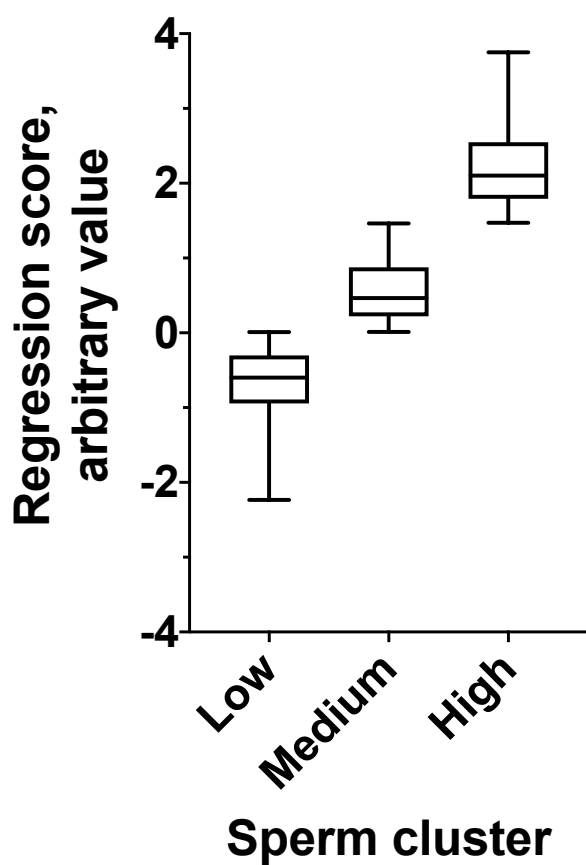

**Neutral Comet**

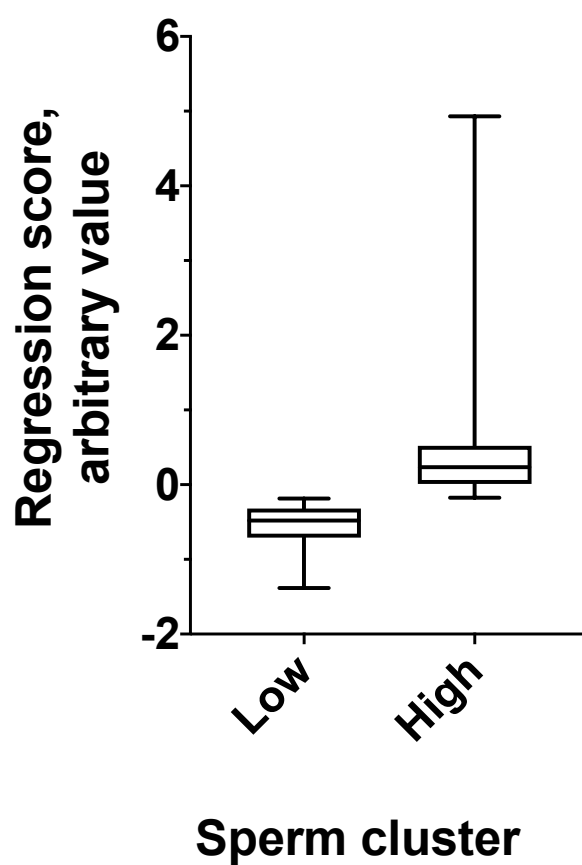

Supplement: Supplementary file 1 — Additional file 1: Fig. S1. Representation of the PCA regression scores in each sperm cluster. Left graph shows sperm with low, medium, or high DNA damage for alkaline Comet. Right graph shows sperm with low or high DNA damage for neutral Comet. [file 40104_2024_1099_MOESM1_ESM.pdf]
