# Supplementary material for: Cryopreservation of bovine sperm causes single-strand DNA breaks that are localized in the toroidal regions of chromatin
Source: J Anim Sci Biotechnol. 2024 Oct 12;15:140. doi: 10.1186/s40104-024-01099-0 (PMC11470689; doi:10.1186/s40104-024-01099-0)
Supplement: Supplementary file 2 — Additional file 2: Table S1. Sperm function parameters in viable and non-viable sperm, before and after cryopreservation. [file 40104_2024_1099_MOESM2_ESM.docx]

**Table S1.** Sperm function parameters in viable and non-viable sperm, before and after cryopreservation.

|  | **Before cryopreservation** | | | | **After cryopreservation**  (mean ± SD) | | | ***P*-value** | | |
| --- | --- | --- | --- | --- | --- | --- | --- | --- | --- | --- |
|  | (mean ± SD) | | |  |  |  |  |  |  |  |
| ***Viability*** |  |  |  |  |  |  |  |  | | |
| Viable sperm (SYBR-14^+^/PI^-^) | 82.54% | ± | 8% |  | 43.51% | ± | 10% | 0.003 | | |
| ***Acrosome integrity*** |  |  |  |  |  |  |  |  | | |
| Viable sperm with an intact acrosome (PNA^-^/PI^-^) | 83.45% | ± | 7% |  | 27.62% | ± | 7% | 0.003 | | |
| ***Membrane lipid disorder*** |  |  |  |  |  |  |  |  | | |
| Viable sperm with high membrane disorder (M540^+^/YO-PRO-1^-^) | 5.75% | ± | 1% |  | 6.83% | ± | 3% | 0.229 | | |
| Viable sperm with low membrane disorder (M540^-^/YO-PRO-1^-^) | 84.35% | ± | 7% |  | 42.52% | ± | 11% | 0.003 | | |
| Non-viable sperm with high membrane disorder (M540^+^/YO-PRO-1^+^) | 9.70% | ± | 7% |  | 50.44% | ± | 13% | 0.003 | | |
| Non-viable sperm with low membrane disorder (M540^-^/YO-PRO-1^+^) | 0.21% | ± | 1% |  | 0.21% | ± | 0% | 0.655 | | |
| ***Intracellular superoxides*** |  |  |  |  |  |  |  |  | | |
| Viable sperm with high intracellular superoxides (E^+^/YO-PRO-1^-^) | 0.20% | ± | 1% |  | 0.00% | ± | 0% | 0.317 | | |
| Viable sperm with low intracellular superoxides (E^-^/YO-PRO-1^-^) | 74.21% | ± | 16% |  | 37.88% | ± | 10% | 0.003 | | |
| Non-viable sperm with intracellular superoxides (E^+^/YO-PRO-1^+^) | 21.22% | ± | 17% |  | 53.64% | ± | 13% | 0.003 | | |
| Non-viable sperm with low intracellular superoxides (E^-^/YO-PRO-1^+^) | 4.36% | ± | 2% |  | 8.48% | ± | 2% | 0.005 | | |
| ***Total ROS*** |  |  |  |  |  |  |  |  | | |
| Viable sperm with high ROS levels (DCF^+^/PI^-^) | 0.09% | ± | 0% |  | 0.67% | ± | 0% | 0.014 | | |
| Viable sperm with low ROS levels (DCF^-^/PI^-^) | 79.88% | ± | 8% |  | 34.12% | ± | 11% | 0.003 | | |
| Non-viable sperm with high ROS levels (DCF^+^/PI^+^) | 0.01% | ± | 0% |  | 0.39% | ± | 0% | 0.317 | | |
| Non-viable sperm with low ROS levels (DCF^-^/PI^+^) | 20.02% | ± | 8% |  | 64.82% | ± | 10% | 0.003 | | |
| ***Intracellular calcium*** |  |  |  |  |  |  |  |  | | |
| Viable sperm with high calcium levels (Fluo4^+^/PI^-^) | 0.31% | ± | 1% |  | 2.27% | ± | 1% | 0.005 | | |
| Viable sperm with low calcium levels (Fluo4^-^/PI^-^) | 81.29% | ± | 14% |  | 27.97% | ± | 9% | 0.003 | | |
| Non-viable sperm with high calcium levels (Fluo4^+^/PI^+^) | 4.34% | ± | 11% |  | 1.52% | ± | 1% | 0.383 | | |
| Non-viable sperm with low calcium levels (Fluo4^-^/PI^+^) | 14.07% | ± | 6% |  | 68.24% | ± | 9% | 0.003 | | |
| ***Mitochondrial activity*** |  |  |  |  |  |  |  |  | | |
| Viable sperm with high mitochondrial membrane potential (JC-1^+^/LD^-^) | 59.73% | ± | 13% |  | 25.63% | ± | 9% | 0.002 | | |
| Viable sperm with low mitochondrial membrane potential (JC-1^-^/LD^-^) | 36.79% | ± | 11% | | 45.18% | ± | 7% | | 0.050 |  |
| Dead sperm with high mitochondrial membrane potential (JC-1^+^/LD^+^) | 0.01% | ± | 0% | | 10.39% | ± | 4% | | 0.002 |  |
| Dead sperm with low mitochondrial membrane potential (JC-1^-^/LD^-^) | 3.48% | ± | 12% | | 18.80% | ± | 6% | | 0.005 |  |

Abbreviations: SD, standard deviation.
